# Novel 4W (When-Where-What-What) Approach of Training Point-of-Care Ultrasound (POCUS) Application in Resuscitation With High-Fidelity Simulator

**DOI:** 10.7759/cureus.9353

**Published:** 2020-07-23

**Authors:** Hong Wang, Adam M Uraco, Justin Stover, Nicole Hollis

**Affiliations:** 1 Anesthesiology, West Virginia University School of Medicine, Morgantown, USA

**Keywords:** point-of-care ultrasound, resuscitation, simulation, application, covid-19

## Abstract

Aim

Point-of-care ultrasound (POCUS) is a valuable tool in anesthesiology used for evaluating and managing cardiopulmonary pathology. Implications of this modality are extensive. Seamless integration into advanced cardiac life support (ACLS) has potential to improve resuscitation outcomes, and there is growing impetus for its implementation during the severe acute respiratory syndrome coronavirus 2 (SARS-CoV-2) pandemic. However, it remains underutilized largely due to limited training. We use high-fidelity simulation and a novel 4W approach (when to apply POCUS, where to place the ultrasound probe, what images mean, what to do next) to incorporate this technique into resuscitation training. This study aims to evaluate the efficacy of a novel 4W POCUS approach for training anesthesiology residents in the setting of resuscitation.

Methods

Our approach teaches learners when and where to implement POCUS, how to interpret their findings, and how to apply these findings in a clinical setting. Learners apply this method in high-fidelity simulation to diagnose and treat cardiopulmonary pathologies. Assessments were administered before and after training to evaluate efficacy.

Results

Post-test improvements were appreciated across all residency classes (n = 23), with achieved significance of P < 0.001 in the first-year clinical anesthesia class (CA-1) and P = 0.02 in the second-year clinical anesthesia class (CA-2). Performance was further subdivided into five categories: resuscitation integration, lung ultrasound, transthoracic echo, disease recognition, and treatment. Post-test scores also improved in each category, with lung ultrasound being the most significant improvement (P = 0.04).

Conclusions

Our initial data demonstrate the effectiveness of this approach to POCUS training. Performance is improved and learners are more likely to use POCUS in the future. The application of this method to larger sample sizes is an appropriate next step to demonstrate its utility.

## Introduction

Intraoperative cardiac arrest occurs in approximately one in 10,000 non-cardiac surgeries [1]. Efficient resuscitation reduces morbidity and mortality and should be reflected in physician training for these scenarios. Point-of-care ultrasound (POCUS) has been successfully used in clinical settings for this purpose [2-5]. POCUS’s implications in acute cardiopulmonary failure are uniquely advantageous by offering quick evaluations without resuscitation interference [6-8]. Similarly, this modality has established new utility amidst the global severe acute respiratory syndrome coronavirus 2 (SARS-CoV-2) pandemic for the evaluation of cardiopulmonary pathology in presumed or confirmed patients [9]. Integration of POCUS into advanced cardiac life support (ACLS) allows concomitant diagnosis and intervention but requires extensive understanding of its indications and timing. Limited training for integrating POCUS into resuscitation remains a barrier to its utilization [10,11]. In response, we have developed a novel approach to POCUS training for anesthesiology residents mimicking real-life scenarios through high-fidelity simulation using the 4W approach (*when* to apply POCUS, *where* to place ultrasound probe, *what* images mean, and *what* to do next). Initial results after training demonstrate improved resident performance in POCUS application, image acquisition, and recognition.

## Materials and methods

Scenario

The scenario used is based on a real case. A 67-year-old male with end-stage renal disease presents for left arterial-venous fistula repair performed under monitored anesthesia care (MAC) sedation and regional anesthesia. His medical history is significant for medically managed mild coronary artery disease, hypertension, chronic obstructive pulmonary disease (COPD) requiring 2 L O_2_ at night, insulin-dependent type II diabetes, and protein C deficiency. A right subclavian central venous line was placed due to poor peripheral access. A left axillary brachial plexus block using 20 mL 0.5% bupivacaine was performed with ultrasound guidance.

During surgery, the patient moves with incision and receives another 10 mL of bupivacaine to strengthen the block. Sixty minutes into the procedure, the patient becomes hypoxic and hypotensive, and registers decreased end-tidal CO_2_. Electrocardiogram (ECG) remains sinus with mild elevation in heart rate to 80, but no carotid pulse is palpable. The patient is in pulseless electrical activity.

The goal of this scenario is for the trainee to quickly differentiate the etiology of the cardiac arrest using POCUS. Differential diagnoses include local anesthetic systemic toxicity (LAST), pneumothorax, cardiac tamponade, and pulmonary embolism (PE).

POCUS curriculum for anesthesia residents at West Virginia University

The Department of Anesthesiology offers biannual POCUS workshops consisting of a 60-minute lecture followed by three-hour hands-on standardized patient workshops. Learners are provided with instructional material for both the lecture and workshops as well as other handouts to supplement their POCUS knowledge. Additionally, each third-year clinical anesthesia resident (CA-3) completes a two-week mandatory POCUS rotation featuring simulation labs, up to one week in the adult echocardiography lab, live scans with faculty, and perioperative POCUS experiences.

4W POCUS application

POCUS application and examination are provided upon request from the trainee. The trainee determines when to conduct POCUS and where to place the probe. The trainee then interprets the displayed POCUS image and decides what is the appropriate next step. Simulated POCUS images include transthoracic echocardiography, subcostal inferior vena cava, and lung ultrasound.

Simulation training

Training is performed using a ultrasound machine (GE Venue Ultrasound System; GE Healthcare, Wauwatosa, WI) and a high-fidelity echocardiography simulator (CAE Vimedix; CAE Healthcare, Sarasota, FL). Each simulation session involves two clinical anesthesia residents: a junior (CA-1) paired with a senior (CA-2 or CA-3). Most residents have attended departmental POCUS workshops. Two have completed their POCUS rotations. The training session includes orientation to the simulation suite, pre-test, simulation, debriefing, and post-test. Each session is conducted with two faculty and one simulation technician.

Statistical analysis

A paired t-test was performed for statistical analysis. SPSS software Version 25 (IBM Corp., Armonk, NY) was used for computation with a P-value <0.05 assigned for determination of statistical significance.

Ethics and patient consent

As a quality improvement project with no personal identifying information used, this study is exempt from West Virginia University Institutional Review Board.

## Results

All trainees successfully diagnosed PE at the end of the simulation and determined the correct treatment option as a group. Based on the clinical scenario, learners successfully ruled out differential diagnoses, including cardiac tamponade, pneumothorax, and LAST. Pre- and post-test assessments evaluated trainees’ integration of POCUS into resuscitation (when), image acquisition (where), disease-specific image recognition (what), and correct treatment selection (what).

**Figure 1:**
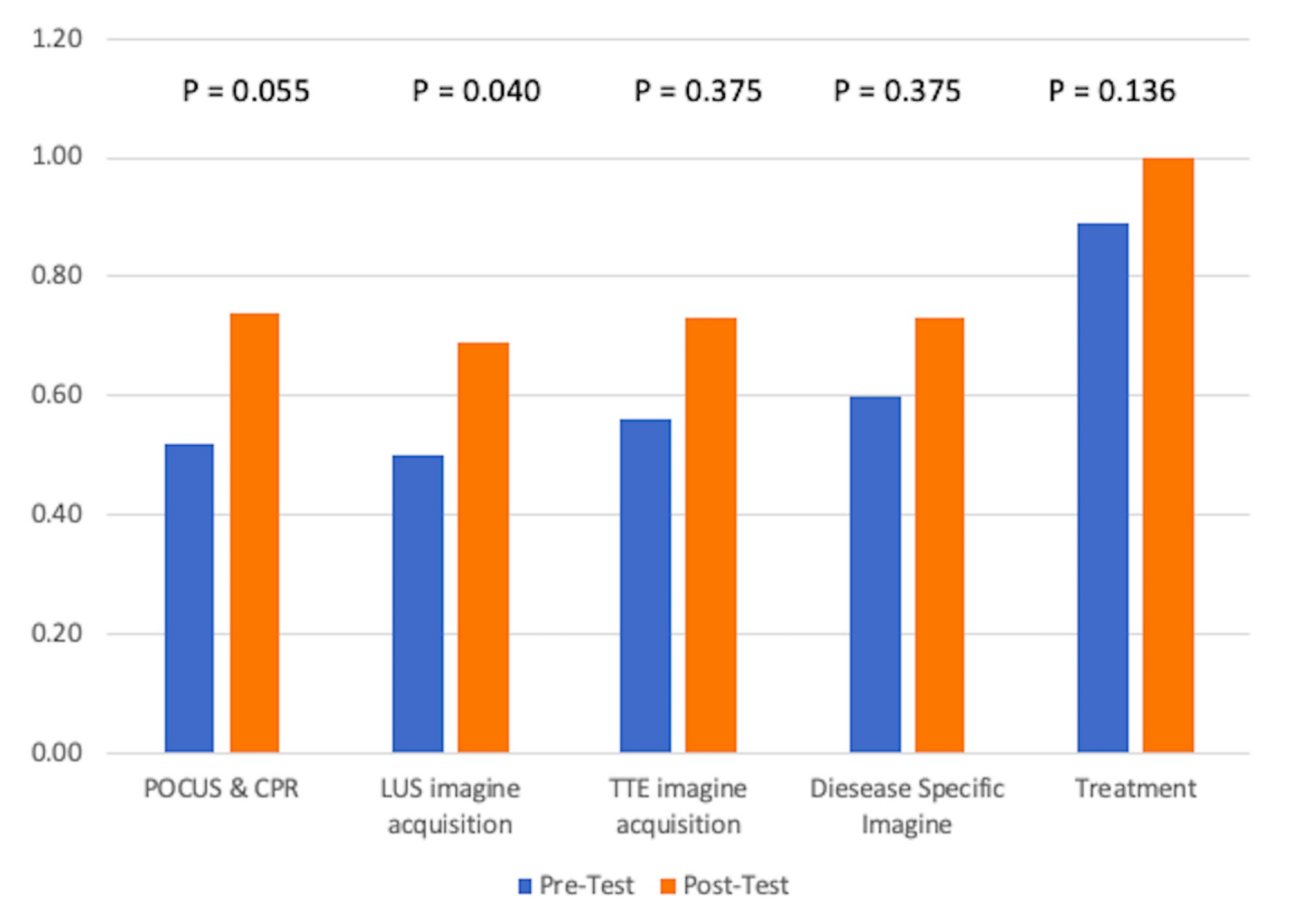
Subject Performance Comparison of pre- and post-test performance for individual subtopics. Scores are expressed in terms of percent correct and represent aggregate data for CA-1, 2, and 3 learners. POCUS = point-of-care ultrasound, CPR = cardiopulmonary resuscitation, LUS = lung ultrasound, TTE = transthoracic echocardiogram.

Performance grouped by subtopics is shown in Figure 1. Pre-test scores were lowest for lung ultrasound and highest for disease treatment. The greatest improvements were seen in lung ultrasound.

Overall, mean post-test scores improved from 55% to 71%. Table 1 shows each class’s improvement. Scores represent overall POCUS performance as a function of percent correct. Data are stratified by residency class. 

**Table 1:** Pre- and Post-Test Score of the Simulation Scores represent overall POCUS performance before and after training as a function of percent correct. Data are stratified by residency class and associated P-values. CA-1 = first-year clinical anesthesia residents (junior residents), CA-2 and 3 = second- and third-year clinical anesthesia residents (senior residents).

	Pre-Test Score	Post-Test Score	Percentage Increase
CA-1	52%	70%	35% (P = 0.0002)
CA-2	55%	71%	30% (P = 0.022)
CA-3	61%	72%	18% (P = 0.124)

All trainees found this POCUS simulation helpful, with 90% more likely to use POCUS in future resuscitations. When surveyed about potential deterrents to future POCUS use in resuscitation, 55% of trainees cite limited training, while 35% endorse limited ultrasound availability. Approximately 65% of trainees believe the simulation is at their training level. 

Data sets used during this study are available from the corresponding author on reasonable request.

## Discussion

POCUS has become a growing topic of anesthesiology research with great focus devoted to evaluating the efficacy of POCUS training. Educational research shows simulation experience to be a positive predictor of success for residents diagnosing cardiopulmonary pathology [12,13]. Following simulation training, learners without previous experience have successfully obtained and interpreted clinical ultrasounds [12]. Similar studies show significant improvements in diagnostic accuracy following the implementation of POCUS protocols into residency curricula [12]. However, the primary emphasis of most approaches continues to be disease-specific image acquisition and recognition [14].

Our initial data corroborated the findings above. Image acquisition and disease recognition improved with POCUS training. However, our approach aims to go beyond image recognition and train residents to ask the question “What's Next?" when performing POCUS. We uniquely integrated POCUS training into a real clinical scenario which included several possibilities based on the patient's history. POCUS was utilized to establish a diagnosis and through the "4W" approach implement the findings into patient care. “When” emphasizes the timing and length of POCUS. It should be performed after the start of chest compressions and mask ventilation. To limit interference with resuscitation efforts and airway management, it should be performed for less than 10 seconds as the role of chest compressions is passed from one provider to the next. “Where” emphasizes what modularity of POCUS should be applied based on the patient’s history and clinical situation. When considering diagnoses, his patient's history of interest included central venous catheter placement concerning for possible pneumothorax or cardiac tamponade, local anesthetic administration potentially resulting in LAST, and a history of coagulopathy concerning for development of PE. “What” emphasizes the user's image interpretation and the second “What” emphasizes how to implement the interpreted diagnosis. We believe this training approach will benefit the trainee to successfully integrate POCUS into perioperative resuscitations. 

To our knowledge, a 4W application-directed approach to simulation emphasizing integration of POCUS into treatment and resuscitation is novel to the field of POCUS training. This approach is necessary for preparing residents for the real-life scenarios they will encounter in practice. This is poignantly illustrated by recent research suggesting that evaluating critical cardiopulmonary scenarios with POCUS is an important step in developing properly tailored resuscitation plans [6,7]. Moreover, when POCUS is incorporated into ACLS and detects cardiac activity, significant improvements in return of spontaneous circulation and hospital survivorship have been observed [6,7].

Similarly, training anesthesiologists in this approach presents the opportunity to improve outcomes surrounding COVID-19 when applied as a means of assessing viral-associated cardiopulmonary pathology. CT has traditionally been used in COVID to screen for pulmonary infiltrates. Although not diagnostic, this technique has been demonstrated as often more sensitive than reverse transcription polymerase chain reaction (RT-PCR) at detecting infection [9]. However, as the pandemic develops, POCUS has emerged as a valid, potentially advantageous proxy to CT for screening and evaluating patients [9]. Due to the highly contagious aerosolized spread of the disease and shortage of personal protective equipment, transporting patients to CT becomes less feasible when bedside lung ultrasound presents as a viable alternative. Not only is POCUS capable of assessing the characteristic lung findings of COVID-19, but it spares patient radiation exposure, implicates less staff in direct patient contact, and is an economically reproducible scan involving equipment which is more easily decontaminated relative to CT [9].

On the whole, our data demonstrate the efficacy of this novel approach. Learners demonstrated improvements in all tested areas, with mean scores increasing in every class. The greatest improvements were amongst CA-1 residents, with slightly less change appreciated in CA-2 and CA-3 classes who began with higher pre-test scores. Their increased baseline performance can be expected as a function of previous POCUS experience. Notably, significant post-test improvements were achieved in lung ultrasound acquisition (P < 0.05). Larger standard deviations contributed to slightly higher P-values in subsequent subjects. The sample size of this study was limited by the number of residents at West Virginia University; however, training larger groups of learners may be an appropriate next step in demonstrating the utility of this application.

## Conclusions

Our initial data demonstrate the effectiveness of this approach to POCUS training. Resident proficiency increased as demonstrated by the post-test performance improvements. Learners additionally are more likely to use POCUS in the future. The application of this method to larger sample sizes is an appropriate next step to demonstrate its utility.

## References

[1] An JX, Zhang LM, Sullivan EA, Guo QL, Williams JP (2011). Intraoperative cardiac arrest during anesthesia: a retrospective study of 218,274 anesthetics undergoing non-cardiac surgery. Chin Med J.

[2] Ellison M, Rangannathan P, Wang H, Vallejo M (2017). Ultrasound and the pregnant patient. Curr Anesthesiol Rep.

[3] Howell S, Bennion D, Jrebi N, Wang H (2018). Application of focused assessment ultrasound in trauma to perioperative medicine: a tool to quickly diagnose postoperative hemorrhage. Anesthesiology.

[4] Hensley J, Wang H (2019). Assessment of volume status during prone spine surgery via a novel point-of-care ultrasound technique. Cureus.

[5] Ranganathan P, Ellison M, Lee JB, Vallejo MC, Wang H (2018). Role of ultrasound in pediatric anesthesia. J Anesth Perioper Med.

[6] Atkinson PR, Beckett N, French J, Banerjee A, Fraser J, Lewis D (2019). Does point-of-care ultrasound use impact resuscitation length, rates of intervention, and clinical outcomes during cardiac arrest? A study from the sonography in hypotension and cardiac arrest in the emergency department (SHoCED) investigators. Cureus.

[7] Blanco P, Martínez Buendía C (2017). Point-of-care ultrasound in cardiopulmonary resuscitation: a concise review. J Ultrasound.

[8] Breitkreutz R, Walcher F, Seeger FH (2017). Focused echocardiographic evaluation in resuscitation management: concept of an advanced life support-conformed algorithm. Crit Care Med.

[9] Buonsenso D, Piano A, Raffaelli F, Bonadia N, DeGaetano Donati K, Franceschi F (2020). Point-of-care lung ultrasound findings in novel coronavirus disease-19 pneumoniae: a case report and
potential applications during COVID-19 outbreak. Eur Rev Med Pharmacol Sci.

[10] Cannon J, Sizemore DC, Zhou Y (2018). Perioperative point of care ultrasound training: a survey of anesthesia academic programs in United States and China. J Anesth Perioper Med.

[11] De Marchi L, Meineri M (2017). POCUS in perioperative medicine: a North American perspective. Crit Ultrasound J.

[12] Parks AR, Atkinson P, Verheul G, Leblanc-Duchin D (2013). Can medical learners achieve point-of-care ultrasound competency using a high-fidelity ultrasound simulator?: A pilot study. Crit Ultrasound J.

[13] Shah S, Tohmasi S, Frisch E (2019). A comparison of simulation versus didactics for teaching ultrasound to swiss medical students. World J Emergency Med.

[14] Parks AR, Verheul G, LeBlanc-Duchin D, Atkinson P (2015). Effect of a point-of-care ultrasound protocol on the diagnostic performance of medical learners during simulated cardiorespiratory scenarios. CJEM.

